# Tuberculosis Exposure from a Healthcare Worker to Patients in a Neonatal Intensive Care Unit (NICU)

**DOI:** 10.1155/2022/2659883

**Published:** 2022-06-29

**Authors:** Maria Casalino, Thivia Jegathesan, Michael Sgro, Elizabeth Rea, Matthew Muller, Douglas M. Campbell

**Affiliations:** ^1^Department of Family Medicine, Queen's University, Kingston, Ontario, Canada; ^2^Department of Pediatrics, Faculty of Medicine, University of Toronto, Toronto, Ontario, Canada; ^3^Division of Clinical Public Health, Dalla Lana School of Public Health, University of Toronto, Toronto, Ontario, Canada; ^4^Division of Infectious Disease, St. Michael's Hospital, Unity Health Toronto, Toronto, Ontario, Canada

## Abstract

The nosocomial spread of *Mycobacterium tuberculosis* from a healthcare worker with infectious pulmonary tuberculosis disease to patients remains a risk in the healthcare environment, including neonatal intensive care units. In this paper, we outlined a protocol for neonates exposed to tuberculosis in a neonatal intensive care unit that includes skin testing, chest X-ray imaging, and prophylactic isoniazid. Neonatal patients were followed up with tuberculosis skin testing at both three months corrected age and two months postexposure. To our knowledge, this is the first Canadian study to illustrate a protocol following tuberculosis exposure in a neonatal intensive care unit for exposed neonates.

## 1. Introduction

Neonatal tuberculosis (TB) disease is rare, but can be a significant cause of mortality, causing death in up to 50% of those infected [1, 2]. It can be acquired haematogenously through the placenta or the umbilical vein, or by aspiration of infected amniotic fluid [1, 2]. Neonatal TB can not only develop as a consequence of airborne respiratory droplets from contact with a close infectious TB source case, typically the mother and/or parent, but may also be transmitted to a neonate from other infectious source cases [3].

Transmission to infants begins with infection and can continue to progress to active disease within weeks to months, without a latent period or reactivation. TB infection occurs when the *Mycobacterium* enters the host and causes an immune response, usually visible in the form of a positive TB test, but the individual may have no signs or symptoms, and/or chest X-ray findings that would suggest active TB disease. TB disease occurs when the infection progresses and the individual exhibits signs and/or symptoms of TB, and chest X-ray findings are suggestive of TB disease. Some studies have outlined nosocomial transmission of TB between healthcare workers and patients. For example, a study in Korea described the transmission of pulmonary TB from 1 nurse to 4 out of 108 infants in a neonatal intensive care unit (NICU) [4]. There are other similar studies that describe neonatal follow-up after exposure to a healthcare worker with infectious pulmonary TB disease [5–8]; however, these studies have had variable outcomes.

Traditionally, a tuberculin skin test (TST) is used to detect TB infection [9]. However, TB follow-up in infants can be complicated by the reliability of tuberculin skin testing [10, 11] as we cannot dependably use TSTs in premature infants exposed to TB. False-negative TST reactions, for example, occur more commonly in infants and children, specifically those younger than 6 months of age [10, 11]. The current recommendation is that TB skin testing should not be routinely conducted before 3 months of corrected age, unless an infant is exposed to tuberculosis [12].

The purpose of this study was to outline a protocol and the outcomes of infants exposed to TB via an infectious healthcare worker in a Canadian NICU setting.

## 2. Methods

**Figure 1:**
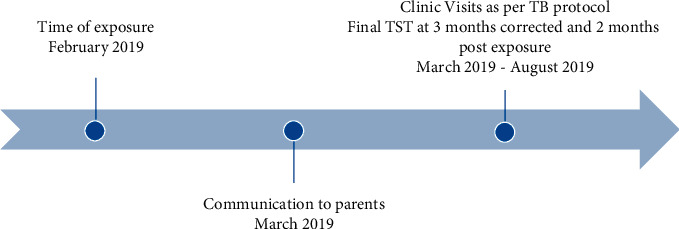
Timeline of exposure through clinical follow-up.

**Figure 2:**
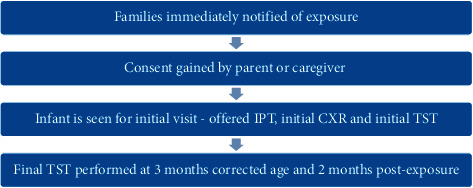
Procedures and methods involved in the study [13].

A retrospective descriptive case series was carried out on infants exposed to a healthcare worker providing direct patient care in the NICU, with active pulmonary TB disease at an academic hospital in Toronto, ON, Canada. The healthcare worker was positive with active tuberculosis disease, as confirmed by a positive culture and sputum smear microscopy for acid fast bacilli. After a careful review of the healthcare worker's clinical duties, the potential exposure period was estimated to be one month. Demographic information of the exposed infants, length and type of exposure to the index case healthcare worker, results of investigations, and six-month follow-up data were collected. Research ethics approval was sought at St. Michael's Hospital. Only infants with close contact with the infected healthcare worker were followed-up. Close contact was defined as all infants that had the healthcare worker as a primary assignment for at least 1 shift (12 hours), or as a secondary assignment (covering for breaks only). Infants exposed as a secondary assignment meant they were in the NICU at the same time as the healthcare worker or received care for only a short period of time if an issue arose.  Figure 1 illustrates a timeline of the clinical follow-up period.  Figure 2 demonstrates the procedures involved in carrying out the study.

### 2.1. Follow-Up Protocol Developed for Exposed Neonates

All families were immediately notified of the exposure. Consent was obtained from parents or caregivers of the exposed infants for ongoing evaluation and follow-up. All exposed infants' parents consented to take part in the study. All infants exposed to TB were followed-up in a make-shift TB clinic at the hospital of the initial exposure. At the time of the clinic visit, the infants were examined, families were provided support, and they were offered the following treatment options:

1. Window isoniazid preventative therapy (IPT) including isoniazid (INH) prophylaxis with a dosing of 10 mg per kilogram per day. Testing included a chest X-ray both anterior-posterior (AP) and lateral of the infant at the first visit, and a TST at three months corrected age and two months postexposure.
2. If the families declined INH prophylaxis for their infants, they were still offered testing. This included a chest X-ray both AP and lateral, a TST at the time of visit, and a TST at three months corrected age and two months postexposure. The TST was used, rather than an interferon-gamma release assay, based on advice from the infectious control team, including paediatric infectious disease specialists. The TST was performed by injecting 0.1 ml of tuberculin purified protein derivative (PPD) into the inner surface of the forearm. An induration of >5 mm was considered indicative of infection.

This multidisciplinary clinic team included staff from pediatrics, radiology, infectious disease, nursing, and public health. The follow-up plan was developed in conjunction with infectious disease and public health as there is limited literature on the follow-up and risks to infants exposed in this manner.

### 2.2. Analysis

Descriptive analysis was used to describe the outcomes of infants exposed to the healthcare worker with TB disease. Outcomes of importance include TB infection, as indicated by a positive TST, or TB disease.

## 3. Results

### 3.1. Demographics

There were 22 infants, 9 females and 13 males, first exposed in our organization between February 1, 2019, and February 20, 2019. At the time of exposure, none of the infants were intubated or ventilated and majority of the infants were held in open cots. In addition, none of the infants received BCG and they were not exposed to or tested for HIV. The maternal HIV status was negative for all. Of the 22 exposed infants, all of them were contacted, offered IPT, and received follow-up at St. Michael's Hospital. Demographic data and infant outcomes can be found in Table 1. The median gestational age at the time of birth was 36 weeks (range 22–38). The median birth weight was 1857 g (range 630–3280). The mean maternal age at the time of birth was 33 years (SD = 5). Of the 20 mothers involved, two had twins, and 14 of the 20 total pregnancies included complications.

### 3.2. Follow-Up Screening and Protocol

Among the infants that attended the follow-up clinic, 19 (86%) received window IPT with INH and Vitamin B_6_. The remaining 3 parents (14%) declined. All the infants underwent AP and lateral chest X-rays, none of which showed features suggestive of TB disease.

### 3.3. Patient Outcomes

The mean hours of exposure via the healthcare worker as a primary assignment was 8 hours (SD = 12). The mean hours of exposure as a secondary assignment was 32 hours (SD = 22). None of the infants exposed developed TB infection or TB disease. A negative CXR indicated that there were no findings suggestive of TB disease. Of the 19 infants that received window IPT, four infants stopped preventative treatment prior to their final TSTs due to perceived feeding intolerance. Of the 21 infants that received a final TST, all 21 tests returned negative for tuberculosis infection. 1 infant passed away prior to receiving a final TST due to complications of prematurity unrelated to tuberculosis or their exposure. The median time from initial assessment to final TST was 29 days (range 17–50).

## 4. Discussion

To our knowledge, this is the second Canadian study to report outcomes of infants in a NICU being exposed to TB from a healthcare worker with infectious pulmonary TB [8]. However, this is the first Canadian study to report a protocol strategy following exposure to tuberculosis. In our case series of 22 infants, none of the infants developed evidence of TB infection or disease despite close contact with the healthcare worker. Our study differs from one study in Korea, where 4 of 108 infants developed TB infection from their exposure [4]. Our study describes a concise follow-up protocol employed for infants exposed to TB, including isoniazid preventative therapy, chest X-rays, and TSTs for exposed infants. Previous studies lacked this combination.

Exposure from a healthcare worker with infectious TB to an infant has rarely been reported in the premature population [5–8]. Only one study from South Korea, to our knowledge, has reported nosocomial transmission of tuberculosis infection to neonates exposed to an infectious healthcare worker [4]. The remaining four studies' reported outcomes of infants exposed to TB from a healthcare worker reported no active tuberculosis infection or disease identified in any of the exposed infants [5–8]. Our study incorporates the use of both AP and lateral chest X-rays at the first visit, while the previous Canada study did not [8]. Other studies differed from ours in that one did not clinically evaluate or empirically treat infants [5], one study did not clarify the type of chest-X-ray used [6], and one did not include any imaging in their follow-up [7]. All of the above studies reported no positive tuberculosis cases following nosocomial exposure from an infected healthcare worker. Premature infants and infants in a NICU with acute illnesses are relatively immune-compromised and at increased risk for severe neonatal complications and bacterial infections [14]. In terms of TB specifically, infected infants are 5–10 times more likely than adults to develop active disease, which can be explained by multiple immune factors that are present as a neonate [14]. In neonates, the number and function of key immune cells, including neutrophils, alveolar macrophages, and dendritic cells, is decreased [14]. Additionally, the pro-inflammatory response in newborns is dampened, while the anti-inflammatory response is increased [14]. This combination explains the vulnerability to active TB disease in the neonatal period.

The study's main limitation is the small number of infants that were exposed. We limited our definition of an exposed infant to only infants that were cared for directly by the affected healthcare worker, either as a primary assignment or secondary assignment. To our knowledge, no other individuals within the neonatal intensive care unit staff or neonates developed TB. We did have a complete follow-up of all exposed infants aside from the one infant that passed away of causes related to prematurity rather than TB. Other limitations include assumptions regarding hours of exposure as primary and secondary assignments, and the full information regarding the healthcare worker's TB course, including drug susceptibility and disease specifics. In addition, the true window of exposure of the healthcare worker was unknown, but estimated to be one month as per infection controls specialist knowledge of the presenting healthcare worker's tuberculosis illness.

One factor in the completeness of follow-up and cooperation of all families is likely related to the prompt protocol and communication immediately upon awareness that a healthcare worker in the NICU had active pulmonary TB disease. This highlights the importance of clear communication and the rapid development of a standardized protocol for follow-up in order to ensure compliance and trust with families. There is still limited literature on the risk of either developing TB or the conversion to skin test positive in neonates exposed to TB by a healthcare worker in a NICU. This is the first Canadian study to our knowledge that describes a protocol following nosocomial TB exposure in a NICU that includes both anterior-posterior and lateral chest X-rays, isoniazid preventative therapy, and follow-up TSTs.

## Figures and Tables

**Table 1:** Exposed infant and maternal demographics and infant outcomes.

Infant demographics	Median (range)
Gestational age (GA weeks)	36 (22–38)
Birth weight (g)	1857 (630–3280)
Discharge weight (g)	2739 (1726–4112)

Maternal demographics	Mean (SD)
Maternal age (years)	33 (5)

Outcomes	Number of infants (%)
Negative chest X-ray*∗*	22 (100)
IPT received	19 (86)
IPT never received	3 (13)
Not on IPT at the time of final TST	10 (45)
Infant death prior to the final TST	1 (5)
Negative final TST result	21 (95)

*∗*Negative chest X-ray, performed at initial screening = no findings suggestive of TB disease.

## Data Availability

Data were collected by chart review and are not part of a publicly archived dataset.
